# Engineering skeletal muscle tissues with advanced maturity improves synapse formation with human induced pluripotent stem cell-derived motor neurons

**DOI:** 10.1063/5.0054984

**Published:** 2021-07-13

**Authors:** Jeffrey W. Santoso, Xiling Li, Divya Gupta, Gio C. Suh, Eric Hendricks, Shaoyu Lin, Sarah Perry, Justin K. Ichida, Dion Dickman, Megan L. McCain

**Affiliations:** 1 Laboratory for Living Systems Engineering, Department of Biomedical Engineering, USC Viterbi School of Engineering, University of Southern California, Los Angeles, California 90089, USA; 2 Department of Biological Sciences, Dornsife College of Arts and Letters, University of Southern California, Los Angeles, California 90089, USA; 3 Department of Stem Cell Biology and Regenerative Medicine, Keck School of Medicine of USC, University of Southern California, Los Angeles, California 90033, USA

## Abstract

To develop effective cures for neuromuscular diseases, human-relevant *in vitro* models of neuromuscular tissues are critically needed to probe disease mechanisms on a cellular and molecular level. However, previous attempts to co-culture motor neurons and skeletal muscle have resulted in relatively immature neuromuscular junctions (NMJs). In this study, NMJs formed by human induced pluripotent stem cell (hiPSC)-derived motor neurons were improved by optimizing the maturity of the co-cultured muscle tissue. First, muscle tissues engineered from the C2C12 mouse myoblast cell line, cryopreserved primary human myoblasts, and freshly isolated primary chick myoblasts on micromolded gelatin hydrogels were compared. After three weeks, only chick muscle tissues remained stably adhered to hydrogels and exhibited progressive increases in myogenic index and stress generation, approaching values generated by native muscle tissue. After three weeks of co-culture with hiPSC-derived motor neurons, engineered chick muscle tissues formed NMJs with increasing co-localization of pre- and postsynaptic markers as well as increased frequency and magnitude of synaptic activity, surpassing structural and functional maturity of previous *in vitro* models. Engineered chick muscle tissues also demonstrated increased expression of genes related to sarcomere maturation and innervation over time, revealing new insights into the molecular pathways that likely contribute to enhanced NMJ formation. These approaches for engineering advanced neuromuscular tissues with relatively mature NMJs and interrogating their structure and function have many applications in neuromuscular disease modeling and drug development.

## INTRODUCTION

Neuromuscular diseases cause progressive muscular atrophy and motor system impairment and affect 160 per 100 000 people worldwide.^1^ These disorders exhibit widely varying inherited^2^ or acquired etiologies^3,4^ and are often associated with neuromuscular junction (NMJ) dysfunction. NMJs are the synapses between motor neurons and skeletal muscle fibers, where acetylcholine released by motor neurons diffuses across the synaptic cleft binds to acetylcholine receptors on muscle fibers and initiates contraction. Most neuromuscular diseases remain incurable^5^ due largely to the complexity of these diseases, which is compounded by a lack of suitable model systems for efficiently and reproducibly investigating human disease mechanisms across multiple spatial scales. For example, transgenic mice have historically been the gold standard for neuromuscular disease modeling,^6,7^ but they cannot recapitulate all human genotypes or phenotypes^7,8^ and are impractical for robustly evaluating the structure and function of neuromuscular tissues on a cellular and molecular level. To investigate neuromuscular tissues at this spatial scale, embryonic rat spinal cord and dorsal root ganglia have been explanted onto cultured primary human muscle tissues to form relatively mature NMJs that are stable *in vitro* for several weeks or longer.^9–11^ However, spinal cord explants consist of multiple cell types, cannot be expanded *in vitro*, and cannot be practically isolated from humans, which limits their reproducibility, scalability, and human relevance, respectively. Thus, there is a pressing need for new patient-specific model systems that enable rigorous examination of neuromuscular disease mechanisms on the microscale.

Human induced pluripotent stem cell (hiPSC)-derived motor neurons have recently become a new paradigm for elucidating the impact of disease-relevant mutations on human motor neurons.^12–14^ To leverage these cells to investigate NMJs in normal and pathological contexts, multiple approaches for co-culturing motor neurons and skeletal muscle have been attempted,^15^ including seeding motor neurons on myotubes^16,17^ and isolating cell types into microfabricated compartments.^18–20^ However, most NMJs *in vitro* consistently fail to recapitulate the mature, pretzel-like structure of native NMJs and instead present patchy, blot-like structures.^16–20^ One potential reason may be that most co-cultured tissues cannot survive beyond two weeks, which is insufficient for NMJ maturation.^12,21^ The maturity of the engineered muscle tissue itself is likely also a bottleneck, which can vary widely depending on myoblast source and culture conditions.^22^ Most *in vitro* approaches have also insufficiently quantified important functional outputs, such as muscle contractility and synaptic activity, due to poor muscle development and/or technological restrictions.^23–25^ Compounded, these limitations have hindered the systematic formation, maturation, and evaluation of NMJs between hiPSC-derived motor neurons and muscle cells *in vitro*.

We hypothesized that NMJs formed by hiPSC-derived motor neurons could be improved by optimizing the maturity of the co-cultured muscle tissue. To test this, we compared the structure and function of muscle tissues engineered from three types of myoblasts: immortalized C2C12 mouse myoblasts, freshly isolated primary chick myoblasts, and commercially available cryopreserved primary human myoblasts. We implemented micromolded gelatin hydrogels as culture substrates because these have previously been shown to improve the culture lifetime, alignment, and maturation of C2C12 myotubes.^23,26,27^ We then co-cultured engineered muscle tissues with hiPSC-derived motor neurons and assessed NMJ structure and function. Chick tissues formed the most mature muscle tissues and NMJs, suggesting that muscle tissue maturity was a critical prerequisite for advanced synapse formation. We also performed bulk RNA sequencing (RNAseq) on engineered chick muscle tissues and identified the activation of gene networks that promote sarcomere organization and innervation of muscle, providing a blueprint of the molecular factors that likely contributed to enhanced maturation and NMJ formation. Collectively, our data demonstrate that NMJ formation by hiPSC-derived motor neurons can be enhanced by improving the maturity of the co-cultured engineered muscle tissue, an approach that can be extended to uncover patient-specific mechanisms of many neuromuscular diseases on the molecular and cellular level.

## RESULTS

### Structure of engineered muscle tissues

**FIG. 1.**
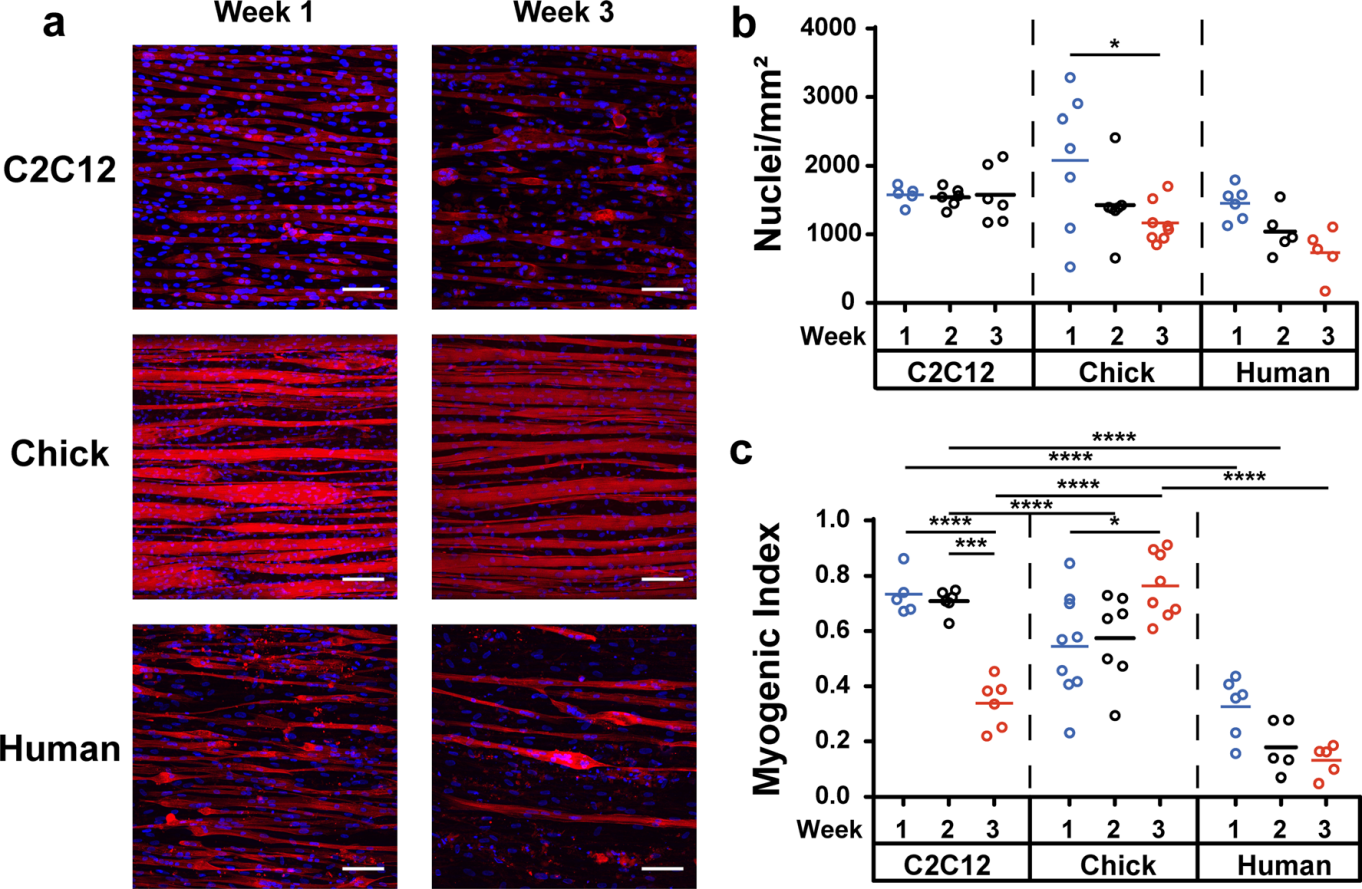
Structure of engineered muscle tissues over three weeks of differentiation. (a) Representative images of C2C12, chick, and human muscle tissues after one and three weeks in culture on micromolded gelatin hydrogels. α-actinin (red), DAPI (blue). Scale bar: 100 *μ*m. (b) Number of nuclei normalized to field of view area and (c) myogenic index for each myoblast cell source at weekly timepoints. ^*^p < 0.05; ^**^p < 0.01; ^***^p <0.001; ^****^p < 0.0001.

Our first goal was to identify a source of myoblasts that yields relatively mature muscle tissues that are stable in culture for at least three weeks. We differentiated muscle tissues from the C2C12 myoblast cell line, cryopreserved primary human skeletal myoblasts, and freshly isolated primary chick myoblasts on micromolded gelatin hydrogels, a substrate previously shown to improve the alignment, maturation, and culture lifetime of C2C12 myotubes.^23,28^ Aligned myotubes were detected for all myoblasts after one week [Fig. 1(a)], but noticeably fewer C2C12 and human myotubes were present after three weeks. To quantify this, we calculated the number of nuclei [Fig. 1(b)], which is expected to be relatively constant over time because myoblasts should continue to fuse, but not proliferate, post-differentiation. Increases in nuclei number could indicate high levels of non-differentiated myoblasts or fibroblasts, which could displace myotubes. Decreases in nuclei number likely indicate cell detachment. In C2C12 muscle tissues, nuclei number remained relatively constant over time. In chick muscle tissues, nuclei number was significantly higher at one week compared to three weeks, which can be largely attributed to the wide distribution of myoblast density after one week due to the natural variation in myoblast purity after cell harvesting. Lower variability at later timepoints suggests stabilization of the tissue. Nuclei number in human muscle tissues decreased with time, although without statistical significance. At each time point, there were no significant differences between cell sources.

To compare myoblast fusion and myotube adhesion, we next calculated myogenic index. Myogenic index for C2C12 muscle tissues was relatively high at one week but declined by three weeks [Fig. 1(c)]. Myogenic index for human muscle tissues started relatively low and trended downwards with time. In contrast, myogenic index for chick muscle tissues increased over three weeks and was significantly higher than C2C12 and human muscle tissues. Thus, only chick myoblasts formed muscle tissues densely packed with aligned myotubes that underwent ongoing fusion over three weeks with minimal detachment.

**FIG. 2.**
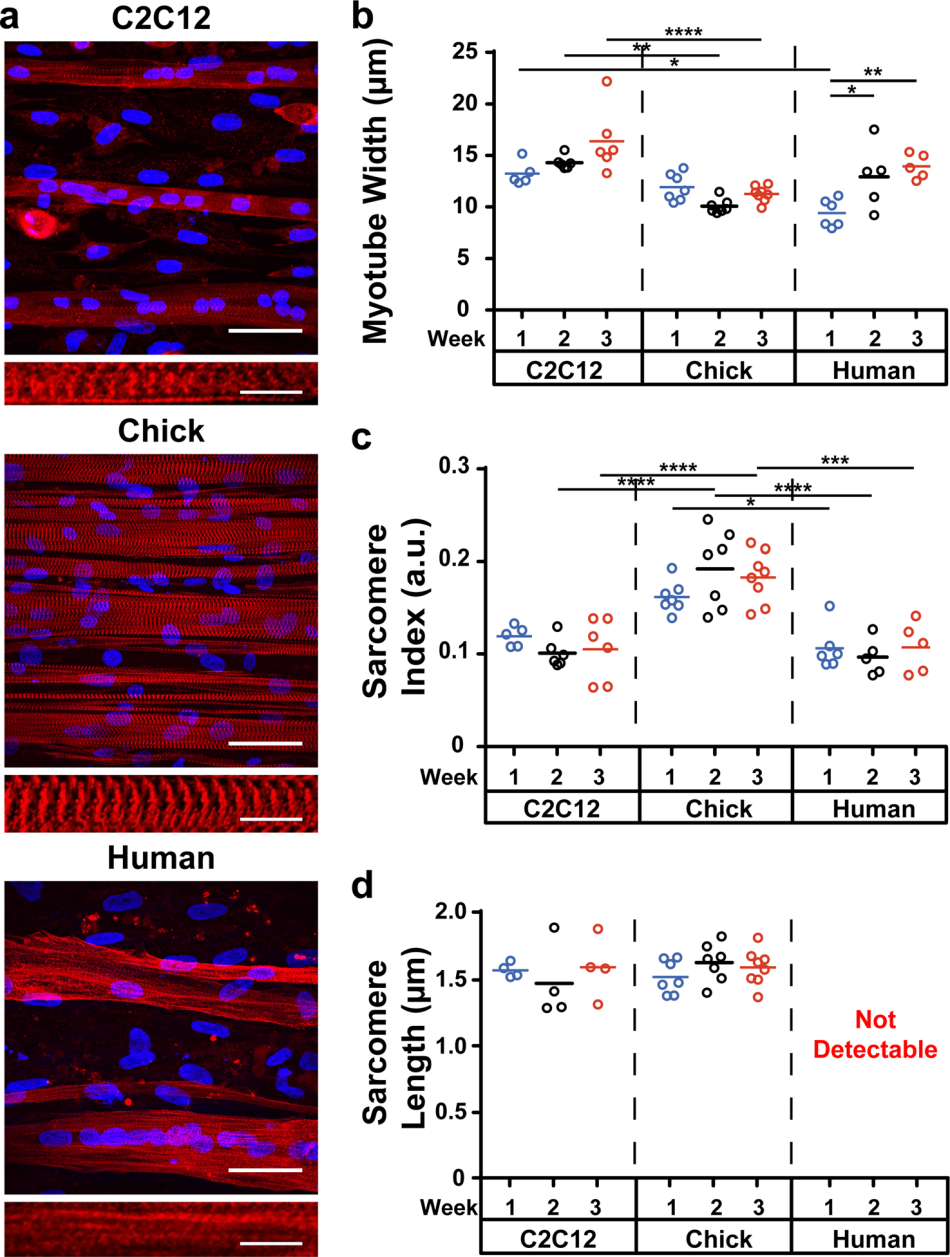
Myotube and sarcomere structure in engineered muscle tissues over three weeks of differentiation. (a) Representative images of C2C12, chick, and human myotubes (top) and sarcomeres (bottom). α-actinin (red), DAPI (blue). Scale bar: 50 *μ*m for top images, 10 *μ*m for bottom images. (b) Average myotube width, (c) sarcomere index, and (d) sarcomere length for each myoblast cell source at weekly timepoints. Human myotubes did not produce sarcomeres that could be analyzed for length. ^*^p < 0.05; ^**^p < 0.01; ^***^p < 0.001; ^****^p < 0.0001.

To assess the morphology of individual myotubes and myofibrils, we quantified myotube width, sarcomere index, and sarcomere length [Fig. 2(a)]. In C2C12 and chick muscle tissues, myotube width was relatively constant over time [Fig. 2(b)]. C2C12 myotubes were significantly wider than chick myotubes at the three-week timepoint. In human muscle tissues, myotube width increased over time, suggestive of slower myotube fusion compared to the other cell sources. However, these increases in width were often associated with increased vacuolization, indicative of apoptosis.^29^ Sarcomere index, which correlates with the periodicity of sarcomeres, was significantly higher in chick myotubes compared to C2C12 and human myotubes at most timepoints [Fig. 2(c)]. Sarcomere length, which ranges from 1.8 to 2.4 *μ*m in healthy vertebrate tissues,^30^ was similar between C2C12 and chick myotubes and was relatively stable over time [Fig. 2(d)]. Human myotubes failed to produce sarcomeres for reliable length quantification. These data indicate that chick myoblasts are optimal for forming structurally mature myotubes and sarcomeres that are stable in culture for at least three weeks.

### Contractile stresses generated by engineered muscle tissues

**FIG. 3.**
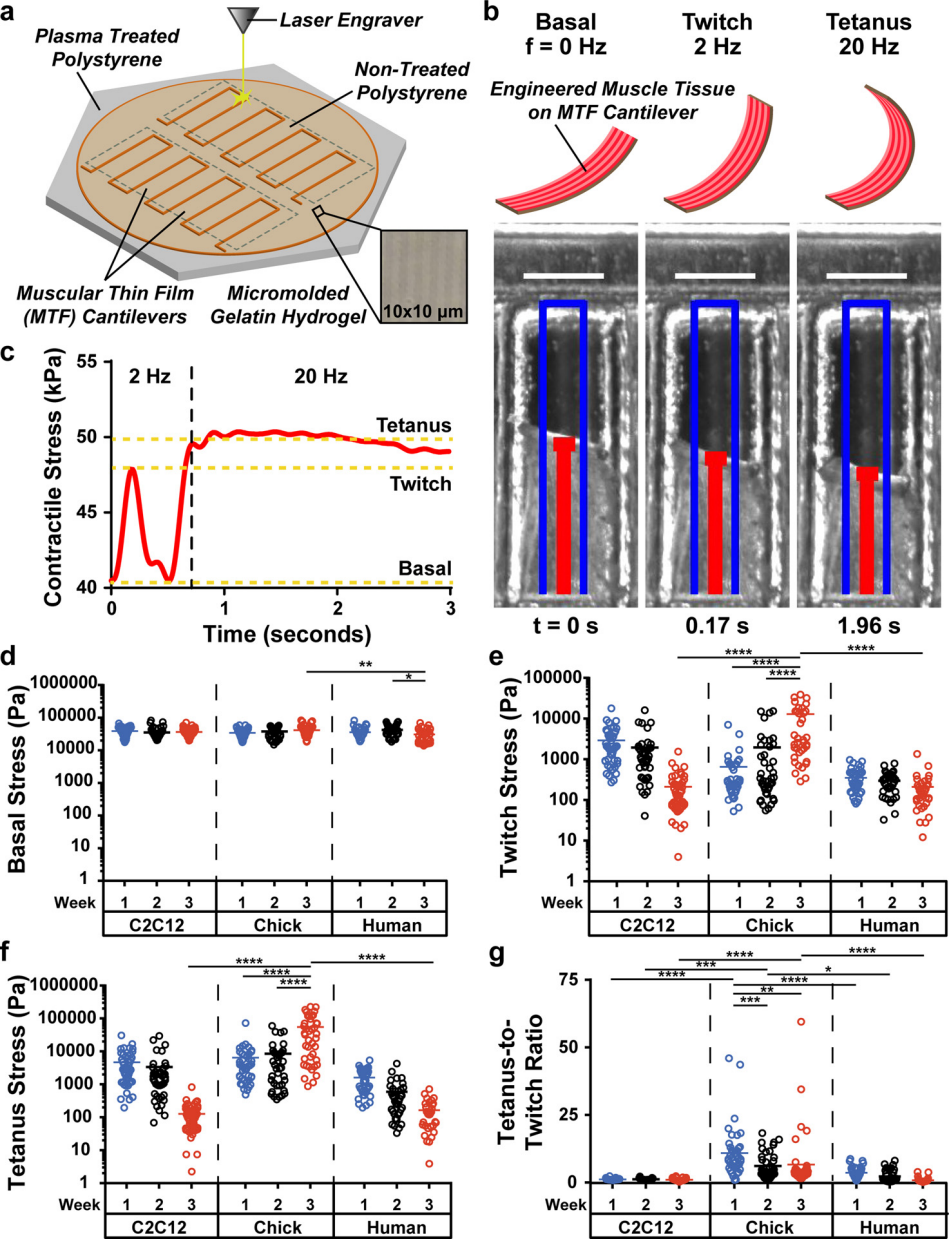
Contractile stresses generated by engineered muscle tissues over three weeks of differentiation, as measured with the MTF assay. (a) Diagram of the fabrication of gelatin hydrogel MTFs. (b) Photographs of a single MTF over time stimulated to contract at twitch and tetanus. Before stimulation (t = 0), the tissue generates basal stress. At 2 Hz stimulation, the tissue generates twitch contractions (t = 0.17 s). At 20 Hz stimulation, the tissue generates a sustained tetanus contraction (t = 1.96 s). Scale bar: 1 mm. (c) Quantification of the stresses generated by the MTF shown in (b). (d) Basal stresses generated by unstimulated MTFs. (e) Twitch stresses generated by stimulating MTFs at 2 Hz. (f) Tetanus stresses generated by stimulating MTFs at 20 Hz. (g) Average tetanus-to-twitch ratios. ^*^p < 0.05; ^**^p < 0.01; ^***^p <0.001; ^****^p < 0.0001.

We next compared the contractility of the engineered muscle tissues. Chick muscle tissues spontaneously contracted approximately 1 day after the onset of differentiation, which continued for about one week. Spontaneous contractions then became infrequent, although bursts of contractile activity were occasionally observed. In contrast, C2C12 and human muscle tissues did not spontaneously contract at any point over three weeks of culture. To quantify differences in contractile stresses, we used the muscular thin film (MTF) assay [Fig. 3(a)] and applied electrical stimulation at 2 and 20 Hz to induce twitch and tetanus contractions, respectively, at weekly timepoints [Fig. 3(b); supplementary material, Video 1]. Basal, twitch, and tetanus stresses [Fig. 3(c)] were calculated based on MTF curvature and the thickness and elastic modulus of the hydrogel, which were measured as 91.3 ± 2.6 *μ*m (n = 6) and 108.3 ± 10.8 kPa (n = 3), respectively. Basal stress was constant across all cell sources and timepoints, except for human muscle tissues at three weeks, which likely had significantly lower basal stress due to myotube detachment [Fig. 3(d)]. For C2C12 and human muscle tissues, twitch and tetanus stresses decreased over three weeks [Figs. 3(e) and 3(f)]. In contrast, twitch and tetanus stresses for chick muscle tissues progressively increased and were significantly higher than C2C12 and human muscle tissues after three weeks. The twitch-to-tetanus ratio, which ranges between 4 and 10 *in situ* and reflects muscle fiber development,^31,32^ was below physiological levels for C2C12 and human muscle tissues [Fig. 3(g)] and was comparable to previous *in vitro* models.^33^ For chick muscle tissues, the twitch-to-tetanus ratio was supraphysiological after one week but fell within physiological ranges by three weeks. At all timepoints, the twitch-to-tetanus ratio was significantly higher in chick muscle tissues compared to C2C12 and human muscle tissues. Together, these data serve as further evidence of enhanced muscle tissue development from chick myoblasts compared to C2C12 and human myoblasts.

### Synaptic structure and activity in engineered muscle tissues co-cultured with hiPSC-derived motor neurons

**FIG. 4.**
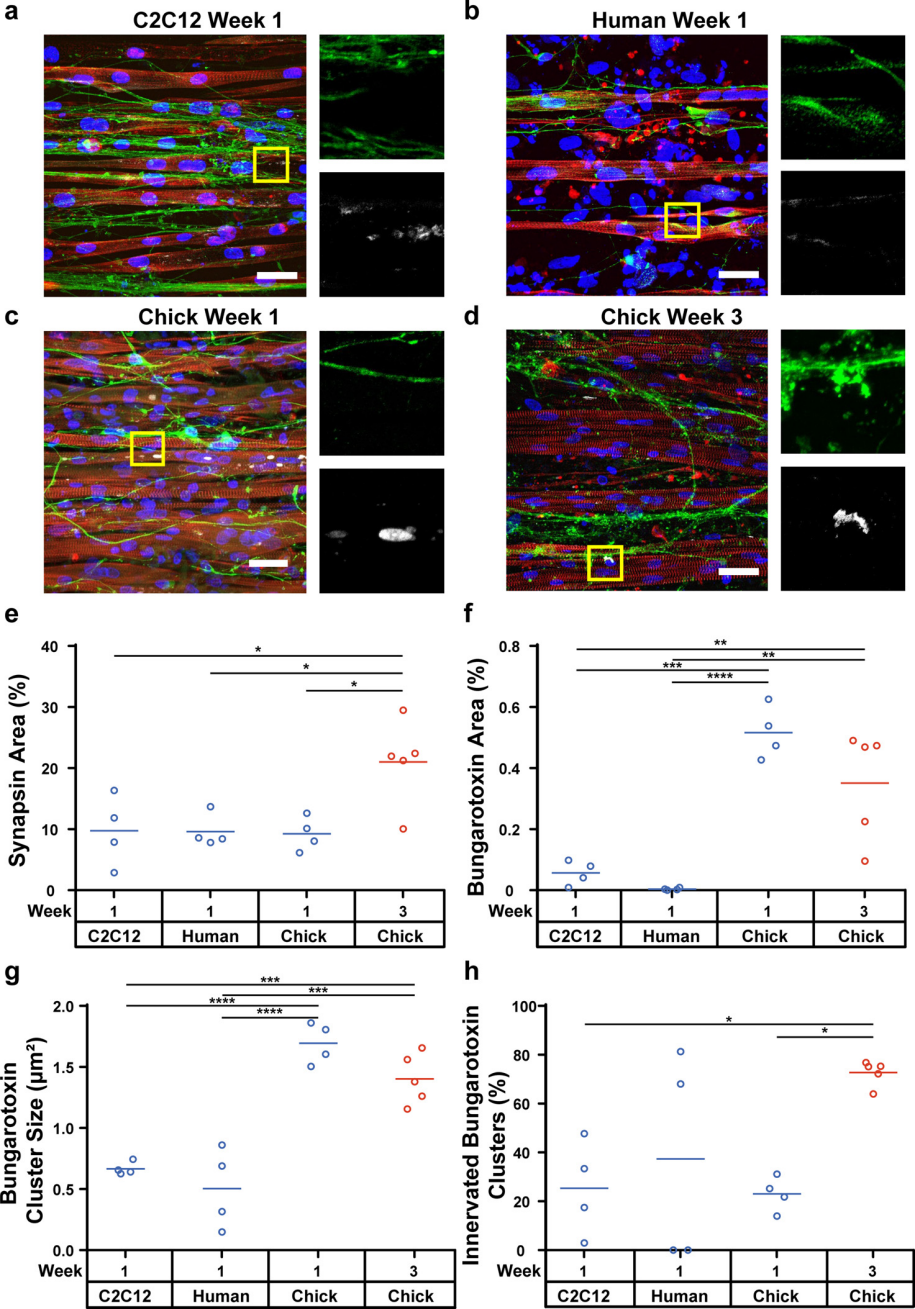
Structure of NMJs formed between engineered muscle tissues and hiPSC-derived motor neurons. hiPSC-derived motor neurons co-cultured with (a) C2C12, (b) human, and (c) chick muscle tissues for one week and (d) chick muscle tissue for three weeks. α-actinin (red), DAPI (blue), synapsin-1 (green), and bungarotoxin (white). Scale bar, 50 *μ*m. Percentage of (e) synapsin and (f) bungarotoxin area per field of view. (g) Size of individual bungarotoxin clusters. (h) Percentage of bungarotoxin clusters co-localized with synapsin. ^*^p < 0.05; ^**^p < 0.01; ^***^p <0.001; ^****^p < 0.0001.

To generate neuromuscular tissues, we next cultured spheroids of hiPSC-derived motor neurons on muscle tissues differentiated from all myoblast sources after 2 to 3 days in differentiation media. Similar to the muscle tissue monocultures, chick tissues spontaneously contracted for several days following motor neuron seeding before contracting sporadically. No spontaneous contractions were observed in C2C12 and human co-cultures at any timepoint. After one week of co-culture, motor neurons extended axons onto myotubes from all myoblast sources [Figs. 4(a)–4(c)]. To evaluate NMJ formation, we quantified the area and co-localization of synapsin and bungarotoxin as pre- and postsynaptic markers, respectively. Synapsin area was similar for all tissues after one week [Fig. 4(e)], indicating similar levels of motor neuron adhesion and spreading. In contrast, the area [Fig. 4(f)] and cluster size [Fig. 4(g)] of bungarotoxin were significantly higher for chick tissues compared to C2C12 and human tissues. Bungarotoxin clusters were barely present in muscle tissue monocultures for all myoblast cell sources (Fig. S1), suggesting that formation of these structures is promoted and maintained by motor neurons. However, even in co-cultured tissues, bungarotoxin clusters did not strongly co-localize with synapsin for any muscle tissue after one week [Fig. 4(h)], indicating poor NMJ formation. To promote NMJ formation, we maintained co-cultured tissues from all myoblast sources for three weeks. However, delamination of C2C12 and human myotubes prevented the survival of co-cultured tissues, and thus analysis of NMJ formation, beyond one week. In contrast, chick muscle tissues co-cultured with hiPSC-derived motor neurons were stable for three weeks and displayed evidence of continued NMJ maturation [Fig. 4(d)], including increased synapsin area [Fig. 4(e)] and increased co-localization of synapsin and bungarotoxin clusters [Fig. 4(h)]. The area and cluster size of bungarotoxin slightly decreased from one to three weeks [Figs. 4(f) and 4(g)], which is expected as non-innervated clusters dissociate.

**FIG. 5.**
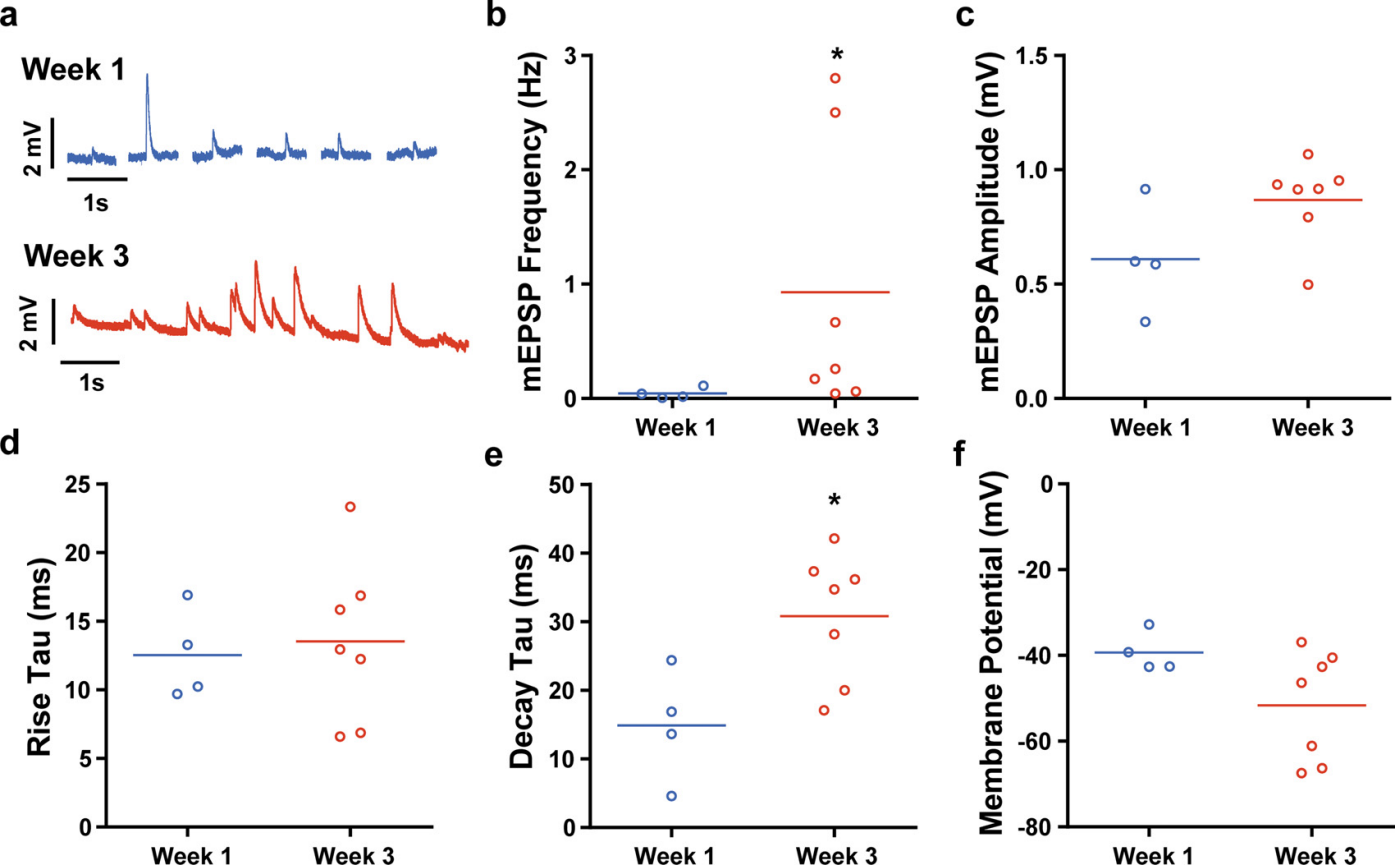
Synaptic activity of NMJs formed between engineered muscle tissues and hiPSC-derived motor neurons. (a) Recordings from representative myotubes after one and three weeks of co-culture with hiPSC-derived motor neurons. (b) mEPSP frequency, (c) amplitude, (d) rise time constant, and (e) decay time constant as a function of co-culture time. (f) Membrane potential of myotubes used for recordings. * denotes p < 0.05.

To evaluate synaptic activity, we performed intracellular sharp electrode electrophysiology experiments^34,35^ to detect miniature excitatory postsynaptic potentials (mEPSPs) in chick muscle tissues co-cultured with hiPSC-derived motor neurons [Fig. 5(a)]. Blebbistatin, a myosin inhibitor, was applied to the tissue to cease spontaneous contractions and motion artifacts that would obscure motor neuron signaling. mEPSPs were more frequent in tissues co-cultured for three weeks compared to one week [Fig. 5(b)], without a significant change in amplitude [Fig. 5(c)]. The average rise time of mEPSPs also did not change from one week to three weeks [Fig. 5(d)], but decay time significantly increased [Fig. 5(e)]. Resting membrane potential decreased slightly from one week to three weeks [Fig. 5(f)], approaching physiological levels of approximately −70 mV.^36^ Together with the structural analysis, these data indicate that improving both the maturity and culture lifetime of the engineered muscle tissue enhanced the development of NMJs with hiPSC-derived motor neurons.

### Transcriptomic analysis of engineered chick muscle tissues

**FIG. 6.**
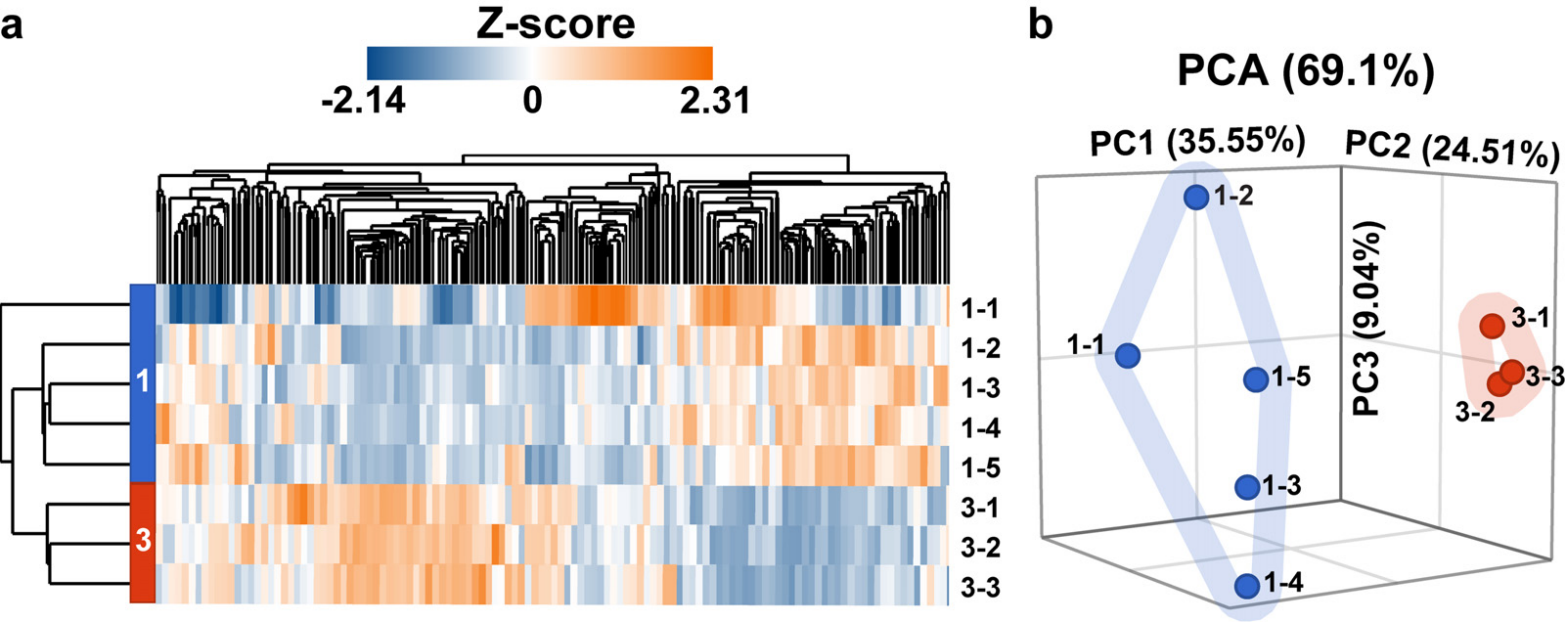
Global transcriptome of chick muscle tissues cultured for one week and three weeks. (a) Hierarchical heat map clustered by features for week 1 (blue) and week 3 (red) samples. (b) PCA plot of week 1 (blue) and week 3 (red) chick tissues. Individual tissues are labeled as “week in culture-sample number” in each panel.

To identify differentially expressed genes (DEG) that correlate with the enhanced maturation and NMJ formation in chick muscle tissues, we performed bulk RNAseq on engineered chick muscle tissues after one and three weeks of differentiation. Figure 6(a) shows a global heat map of the transcriptome of multiple chick tissues after one and three weeks. Principal component analysis (PCA) identified that roughly 69% of the variation in the data could be explained by the first three principal component vectors containing the weighted contributions of each gene in the data set [Fig. 6(b)]. Key genes in the highest 2% of component loading magnitudes are shown in Table I, with upregulation or downregulation at three weeks compared to one week indicated by arrows. Note that the direction of component loading corresponds to gene expression changing in the same direction and is not indicative of up- or downregulation. A complete list of genes and component loadings for eight principal components is in Table S1 and a complete list of fold changes for each gene is in Table S2. Notably, several genes involved in sarcomere structure (*MYH1G*, *TNNI1*, *ACTN1*, *HYAL1*, *DES*),^37,38^ fast/slow fiber phenotype specification (*MYH1G*, *SOX5*),^37,39^ calcium signaling (*CAPN6*, *CAPN10*, *PVALB*),^37^ and mitochondrial respiration (*MB*, *MFN1*, *MFN2*)^37^ have high component loadings, indicating that these structures and processes are actively remodeling in chick muscle tissues with increasing culture time. Intriguingly, synapse markers or other key players in NMJ formation (*AGRN*, *CHRNA1*, *PLEKHG5*)^37^ also had high loading, but were downregulated over time, as discussed in more detail below.

**TABLE I.** Key genes and their component loadings within the first three principal components. Direction of expression fold change is given by arrows. Descriptions of each protein coded by these genes in skeletal muscle tissue are summarized from profiles in the GeneCards database.^37,112^

	Gene	Load	Expression change	Protein description
PC1 (35.55%)	CAPN6	0.99	↓	Calcium-dependent protease suppressing muscle differentiation through regulation of actin reorganization
	MYH1G	−0.98	↑	Chick contractile protein in fast type IIX muscle fibers; similar to human MYH1
	TNNI1	−0.99	↑	Regulatory protein of striated muscle contraction and relaxation
	MB	−0.99	↑	Iron- and oxygen-binding protein for transport in muscle
	ACTN1	−0.99	↑	Sarcomere protein; anchors actin filaments
PC2 (24.51%)	HYAL1	0.88	↑	Hyaluronidase facilitating fusion of myoblasts
	MFN2	−0.95	↓	Mitochondrial membrane protein enabling mitochondrial fusion with initial tethering
	MFN1	−0.97	↓	Mitochondrial membrane protein enabling mitochondrial fusion by membrane integration
	AGRN	−0.97	↓	Glycoprotein central in acetylcholine receptor clustering during NMJ development
	CHRNA1	−0.98	↓	Nicotinic acetylcholine receptors subunit, the main neurotransmitter involved in human muscle function
PC3 (9.04%)	PLEKHG5	0.84	↑	Nucleotide exchange factor regulating autophagy of synaptic vesicles
	DES	0.82	↑	Muscle-specific intermediate filament regulating sarcomere architecture
	PVALB	0.80	↑	Cytosolic calcium ion buffer regulating muscle relaxation
	SOX5	−0.72	↓	Transcription factor maintaining fast myofiber phenotypes
	CAPN10	−0.80	↓	Calcium-dependent protease regulating insulin-stimulated glucose uptake

**FIG. 7.**
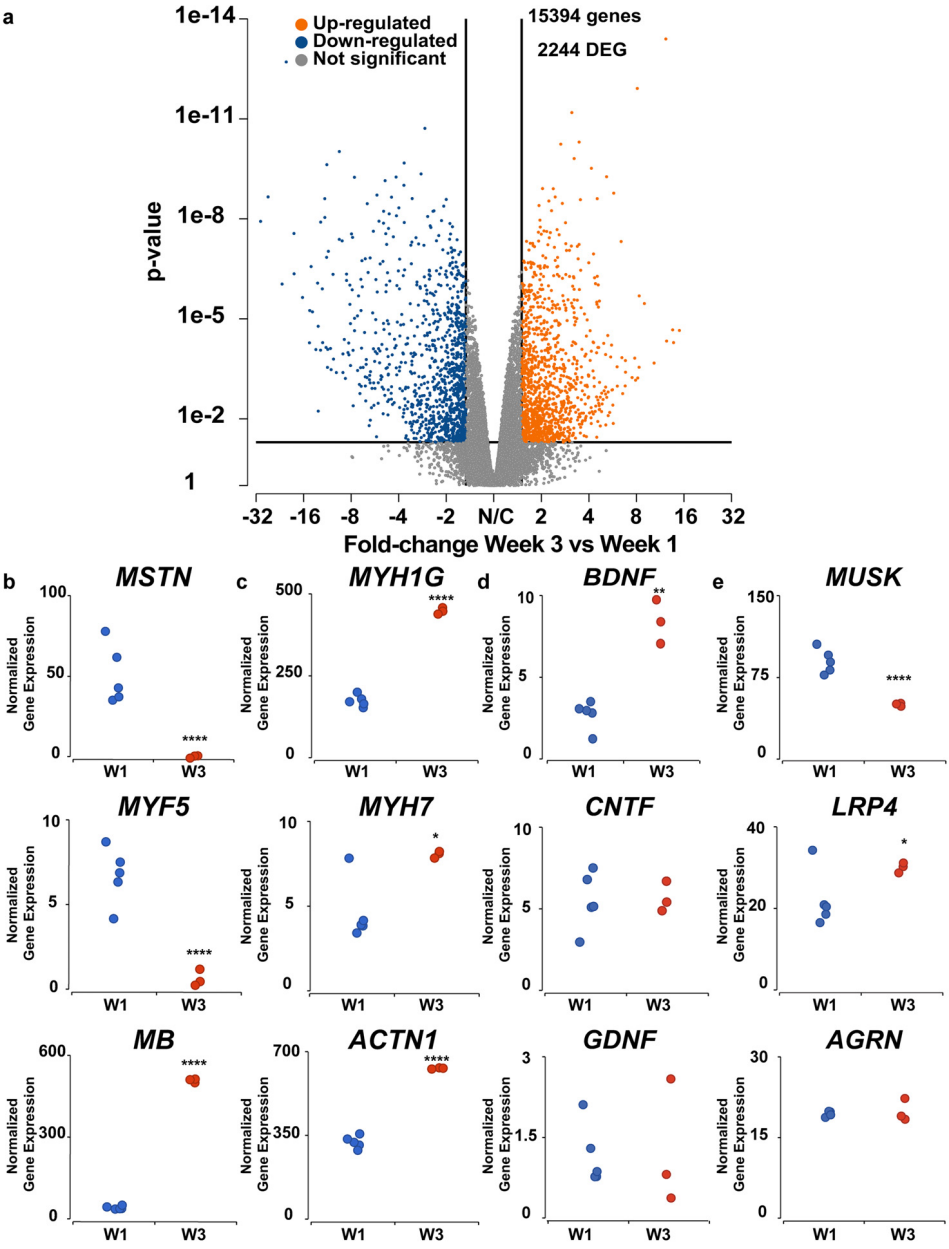
Differentially expressed genes in chick muscle tissues cultured for one week (W1) and three weeks (W3). (a) Volcano plot of all genes up- and downregulated or not significantly changed in tissues cultured for three weeks compared to one week. Differentially expressed genes (DEG) were defined as those with p < 0.05 and a magnitude fold change of greater than 1.5. Normalized gene expression of (b) regulators of myogenic proliferation and metabolism, (c) contractile proteins, (d) neurotrophic factors, and (e) signaling molecules in acetylcholine receptor clustering. *p < 0.05; **p < 0.01; ***p <0.001; ****p < 0.0001.

Next, we performed gene specific analysis between chick muscle tissues cultured for one week and three weeks. As shown by the volcano plot in Fig. 7(a), 2244 of the 15 394 detected genes were differentially expressed. We mined the dataset and identified several differentially expressed genes with known role(s) in muscle development and physiology. Myostatin (*MSTN* or *GDF8*) and myogenic factor 5 (*MYF5*), which are markers of proliferating myoblasts,^40,41^ are downregulated, consistent with increased fusion of myoblasts into myotubes. Myoglobin (*MB*), an oxygen transporter and buffering protein in striated muscle,^37^ exhibited one of the highest fold-changes in normalized gene count (∼12×), possibly to meet the higher mitochondrial respiratory demands of contractile myotubes compared to myoblasts [Fig. 7(b)]. *MB* upregulation is also expected during the maturation of slow-twitch fibers that consume oxygen and expend energy over sustained time periods.^42^ The development of contractile units was also demonstrated by the upregulation of several sarcomere proteins [Fig. 7(c)], including the chicken-specific myosin heavy chain 1G (*MYH1G*, similar to human *MYH1)*, myosin heavy chain 7 (*MYH7*), and alpha-actinin (*ACTN1*). Thus, analysis of muscle-related genes in the bulk transcriptome suggests that changes in aerobic respiration, myoblast proliferation and fusion, and sarcomere development are in part responsible for the structural and functional improvements observed in chick muscle tissues after three weeks in culture.

Due to their role in promoting motor neuron survival,^43^ we also compared the expression of brain-derived neurotrophic factor (*BDNF*), ciliary neurotrophic factor (*CNTF*), and glial cell-derived neurotrophic factor (*GDNF*) [Fig. 7(d)]. Of these three factors, only *BDNF* was significantly upregulated. Finally, we compared the expression of genes related to the AGRN-LRP4-MUSK complex [Fig. 7(e)]. In native NMJs, agrin (AGRN), a proteoglycan secreted by motor neurons that stabilizes the developing synapse, binds to activated complexes of low-density lipoprotein receptor-related protein 4 (LRP4), a sarcolemma receptor for agrin, and muscle associated receptor tyrosine kinase (MUSK), a sarcolemma receptor that congregates LRP4. Motor neuron-derived AGRN binding to the LRP4-MUSK complex clusters acetylcholine receptors,^44,45^ initiating synaptic differentiation. Our data show that *MUSK* is significantly downregulated after three weeks in chick muscle tissues despite an increase in *LRP4*. This downregulation of *MUSK* is likely a response of the muscle tissue to a lack of agrin, which is secreted by motor neurons that are absent in these tissues. Although the chick muscle tissues did express *AGRN*, the amount of AGRN produced by the muscle tissue was likely insufficient to induce acetylcholine receptor clustering. Additionally, compared to neuron-secreted isoforms of AGRN, muscle-secreted AGRN isoforms generally lack an insertion of amino acids at a specific sequence location, referred to as the B site (Z site in mammals). The absence of these amino acid insertions reduces the potency of AGRN to cluster acetylcholine receptors by several magnitudes.^111^

**FIG. 8.**
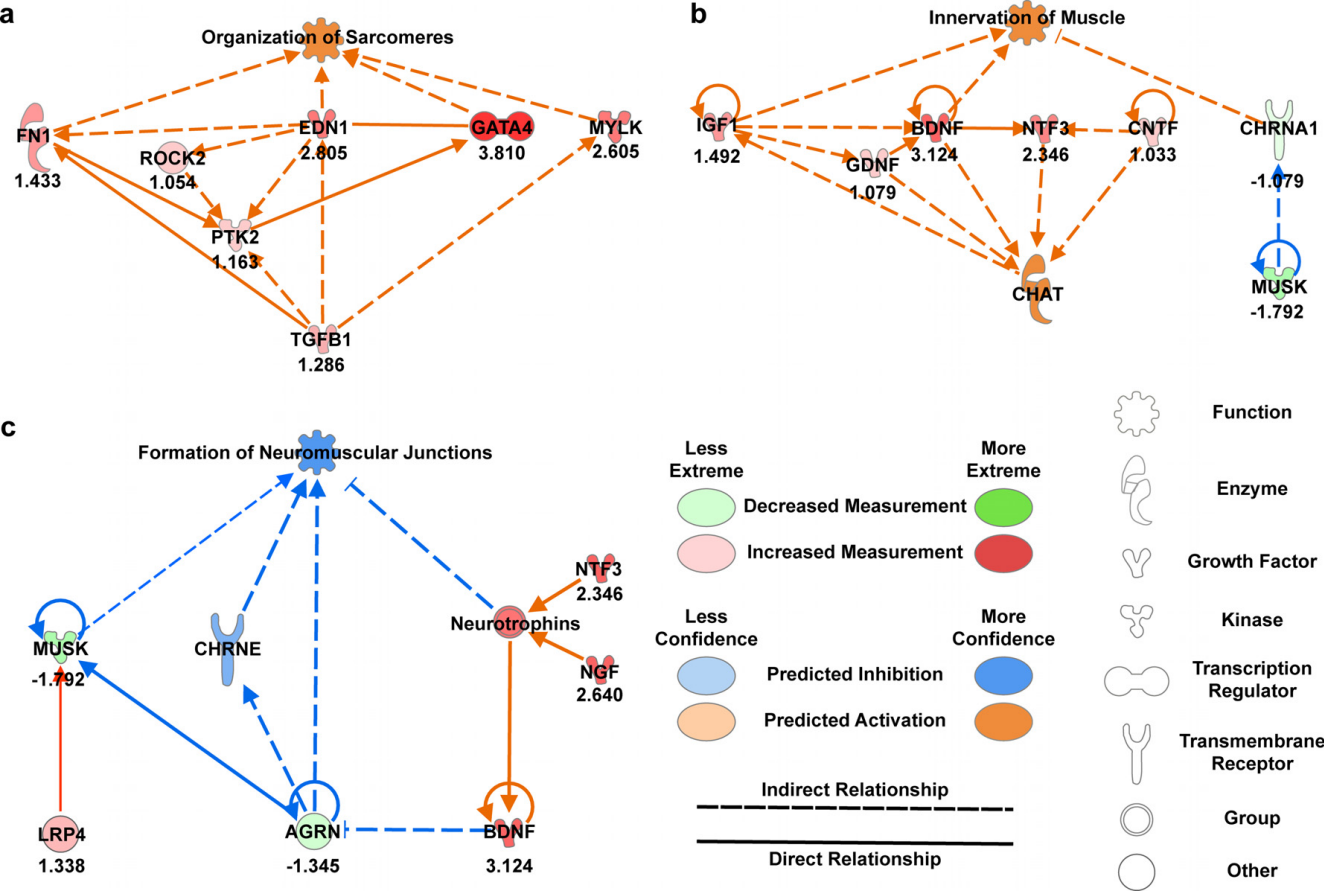
IPA-generated predictive mechanistic networks. In chick muscle tissues cultured for three weeks compared to one week, pathways for (a) organization of sarcomeres and (b) innervation of muscle are predicted to be activated. (c) The pathway for formation of neuromuscular junctions is expected to be inhibited.

Next, we used Ingenuity Pathway Analysis (IPA) to generate predictive mechanistic networks based on the transcriptome data. We found that the network for organization of sarcomeres was predicted to be activated due to upregulation of *FN1*, *ROCK2*, *EDN1*, *GATA4*, *MYLK*, and *TGFB1* [Fig. 8(a)]. The network for innervation of muscle was also predicted to be activated due to an upregulation of neurotrophic factors (*IGF1*, *BDNF*, *CNTF*, and *GDNF*) and a downregulation of *MUSK* [Fig. 8(b)]. Formation of NMJs was predicted to be inhibited based on changes in *BDNF*, *AGRN*, and *MUSK* [Fig. 8(c)], which can likely be attributed to the lack of motor neurons in these tissues. Overall, the transcriptomic data corroborate the structural and functional data and provide additional insights into the expression of genes that promote sarcomere development and innervation in engineered muscle tissues.

## DISCUSSION

Patient-specific modeling of neuromuscular diseases *in vitro* has been limited by the stunted maturation of NMJs formed by hiPSC-derived motor neurons and engineered muscle tissues.^16–18,21^ Here, we improved the structure and function of NMJs formed by hiPSC-derived motor neurons by optimizing the maturity of the engineered muscle tissue. We also identified genes that likely contribute to enhanced muscle tissue maturity and motor neuron integration, providing new insights into the molecular processes that contribute to the development of relatively mature neuromuscular tissues *in vitro*.

Several myoblast sources have been utilized to engineer muscle tissues *in vitro*, including C2C12 myoblasts,^46,47^ primary animal and human myoblasts,^33,48^ and hiPSC-derived myoblasts.^49,50^ However, myotube detachment from conventional synthetic substrates, such as glass or polydimethylsiloxane (PDMS) coated or micropatterned with matrix proteins, prevents survival or maturation beyond approximately two weeks.^23,51^ To overcome myotube detachment, we implemented gelatin hydrogels with 10 *μ*m-wide, 2 *μ*m-deep ridges spaced by 10 *μ*m as culture substrates, based on previous studies showing that these grooved substrates align, mature, and stabilize engineered muscle tissues from C2C12 myoblasts^23,28^ and cardiac myocytes.^52^ The 10 *μ*m topography was chosen to fit the width of an average myotube and yielded aligned myotubes with sarcomere structures similar to myotubes on substrates with nanoscale patterns that match the dimensions of a myofibril^53^ or extracellular matrix proteins.^54,55^ Although micromolded gelatin hydrogels supported the adhesion and alignment of C2C12, chick, and human myotubes after one week in culture, only chick muscle tissues demonstrated increased myogenic index and stable sarcomere index after three weeks. In C2C12 and human muscle tissues, many myotubes detached before the three-week timepoint, limiting their maturation. A different hydrogel topography on the gelatin hydrogels, such as nanoscale grooves,^53–55^ may reduce delamination and promote maturation, but this was not explored in this study.

Especially because they align myotubes, micromolded gelatin hydrogels are compatible with the MTF assay to measure contractile stresses, as shown previously for C2C12^56,57^ and human^58^ muscle tissues. Other assays for evaluating contractile stress in engineered muscle,^59^ such as traction force microscopy^60^ or measuring deflection of micro-cantilevers,^61^ are limited to single myotubes. Using the MTF assay, we found that chick tissues out-performed C2C12 and human tissues and generated contractile stresses in the 100 kPa range, which is similar in magnitude to human tibialis anterior and soleus muscles.^32,62^ In contrast, engineered human, murine, or avian muscle tissues in previous reports generated stresses in the low tens of kPa.^32,63^ In our study, the tetanus-to-twitch ratio of chick tissues was also comparable to *ex vivo* rat^64^ and human muscle tissue,^65^ while C2C12 and human tissues fell between values reported for other previous engineered muscle tissues.^32,66^

In prior attempts to co-culture muscle tissues and primary,^16,67^ embryonic stem cell-derived,^68^ or iPSC-derived^18–20,69^ motor neurons, NMJs were relatively immature, exhibiting low co-localization of pre- and postsynaptic markers^16,21,67,70^ and low frequency of spontaneous or induced synaptic activity^16,17,21^ compared to native muscle.^71,72^ In our co-cultured tissues, hiPSC-derived motor neurons projected axons onto myotubes and several myotubes exhibited clusters of acetylcholine receptors after one week, a pattern observed during embryonic development.^73^ However, similar to previous *in vitro* approaches, most clusters of acetylcholine receptors did not co-localize with axons in any co-cultured tissues after one week, indicating relatively immature NMJs. Co-cultured C2C12 and human tissues detached prior to the three-week timepoint, preventing NMJ maturation. In contrast, hiPSC-derived motor neurons co-cultured with chick tissues continued to extend axons that increasingly co-localized with acetylcholine receptors over three weeks. In parallel, clusters of acetylcholine receptors lacking innervation dissipated, likely because of spontaneous contraction of the chick myotubes. This trend is consistent with reduced acetylcholine receptor clusters in contracting muscle fibers from three-week-old spinal cord explant-muscle co-cultures.^9^ A similar phenomenon has also been observed in NMJ formation during embryonic development.^73^ Spontaneous contractions during early muscle tissue differentiation have also been shown to precede striation of *Drosophila* muscle fibers^74^ and are a key predictor of advanced Z-line development and maintenance of rat myotubes *in vitro*.^75^

To quantify synaptic activity, we performed electrophysiology experiments to detect mEPSPs in chick myotubes co-cultured with hiPSC-derived motor neurons. mEPSPs are generated by spontaneous neural activity and release of neurotransmitter into the NMJ, as shown in *Drosophila*^34,35^ and spinal cord explant-muscle co-cultures.^10^ At the one-week timepoint, our measurements were similar to un-aligned, nine-day co-cultures of C2C12 myotubes and mouse embryonic stem cell-derived motor neurons.^17^ However, at the three-week timepoint, the amplitude and frequency of mEPSPs increased, approaching levels observed in *Drosophila*,^35^ mice,^72^ and spinal cord explant-muscle co-cultures.^11^ The mEPSP decay time also increased from one to three weeks, consistent with an increase in acetylcholine receptors on the myotube membrane that increase the time required for acetylcholine to reach equilibrium, be degraded by acetylcholinesterase, and be reabsorbed into the pre-synaptic terminal. Finally, the membrane potential of innervated myotubes in our system ranged from −40 to −70 mV, roughly the same as myotubes co-cultured with spinal cord explants^11^ and approaching the adult human value of −70 mV.^36^ Thus, our *in vitro* system recapitulated some key steps in the native development of NMJs. However, our systems do fall short in capturing several other important aspects, such as myelinated axon fibers that are in spinal cord explants due to the presence of glial cells.^9,76^ To address this, iPSC-derived glial cells^77^ and astrocytes^78^ could potentially be integrated into our system to advance the maturity and relevance of the NMJs. Additionally, electrophysiology experiments in the presence of drugs known to effect NMJ activity, such as the acetylcholine receptor antagonist tubocurarine, should be performed to verify appropriate physiological responses.

To elucidate the molecular changes that may be responsible for the improved contractile function of engineered chick muscle tissues, we performed RNAseq analysis after one and three weeks of culture to identify genes that are differentially expressed at distinct stages of maturation. We observed downregulation of endogenous myostatin (*MSTN*) and myogenic factor 5 (*MYF5*), which increases expression of the master myogenic regulatory gene *MYOD1*^40^ and decreases myoblast proliferation,^79,80^ respectively. Thus, downregulation of these genes would be expected to push myoblasts to exit the cell cycle, fuse, and differentiate into myotubes. Expression of sarcomere proteins, such as α-actinin (*ACT1*), troponin I (*TNNI1*), and myosin heavy chain (*MYH1G*), was significantly higher after three weeks of culture. While *ACT1* and *TNNI1* expressions are expressed early in embryonic and neonatal myotube development, the increased expression of *MYH1G*, an adult chicken myosin isoform prevalent in fast-twitch type IIx fibers, is indicative of progression toward more mature muscle phenotypes.^25^ This is especially interesting because engineered tissues often exhibit low proportion of fast-twitch fibers.^81,82^ The development of adult slow-twitch fibers was also evidenced by upregulation of slow myosin heavy chain isoform *MYH7*.^83^ However, other mature sarcomere markers, such as myomesins (*MYOM1*, *MYOM2*, *MYOM3*) that are expected to be upregulated^25,84^ and non-muscle myosins (*MYH9*, *MYH10*) that are expected to be downregulated,^25^ remain largely unchanged, indicating that not all sarcomere proteins follow the expected developmental trajectory *in vitro*. However, our Ingenuity Pathway Analysis still showed that, overall, pathways for sarcomere organization are activated in chick muscle tissues cultured for three weeks compared to one week.

We also investigated the expression of genes important for motor neuron survival and integration. The expression of neurotrophic factors *BDNF*, *CNTF*, and *GDNF* was of particular interest because they promote motor neuron differentiation and survival and thus are often added to the differentiation media for hiPSC-derived motor neurons.^85^ Of these factors, *BDNF*, which has been shown to promote innervation of rat diaphragm muscle post-injury,^86^ was significantly upregulated in chick muscle tissues cultured for three weeks. *BDNF* is produced by skeletal muscle in response to contraction^87^ and thus the upregulation of *BDNF* could be caused by the increased contractility of chick muscle tissues at three weeks. Higher levels of BDNF may also enhance lipid oxidation, an adaptation of skeletal muscle to facilitate increased energy expenditure.^88^ However, the timing of *BDNF* expression is important to consider for motor neuron integration, as BDNF also inhibits NMJ maturation after initial innervation.^89^ As shown by our Ingenuity Pathway Analysis, neurotrophins generated by muscle tissue, like BDNF, inhibit *AGRN* expression,^90^ which is needed for activation of the LRP4-MUSK complex and clustering of acetylcholine receptors to form mature NMJs.^44^ In other words, as previously observed,^91^ BDNF is needed for the growth phase of neuromuscular tissues but not the synaptogenesis phase. For this reason, BDNF and other neurotrophic factors are usually removed from culture media after one week of co-culture with muscle tissue. We also observed that *FGF2*, which encodes for fibroblast growth factor 2 (FGF2), was upregulated in chick tissues at three weeks. FGF2 interacts with neuronal receptors, such as fibroblast growth factor receptor 1, to slow axonal growth and promote synapse formation by counterbalancing the growth promoting effects of neurotrophins.^92^ Some genes for key proteins in NMJ formation and maintenance (*AGRN*, *CHRNA1*, and *PLEKHG5*) were downregulated in chick muscle tissues after three weeks. However, this is not too surprising because agrin is primarily secreted by motor neurons to induce acetylcholine receptor clustering^93^ and the tissues used for RNAseq were muscle tissues without motor neurons. Thus, the activation of genes important for NMJ formation and stabilization in muscle is likely dependent on the presence of motor neurons.

Although chick muscle tissues were optimal for NMJ formation and function, a major drawback is their non-human origin. The only human myoblasts tested in this study had relatively low myotube formation and stability, precluding robust NMJ formation. However, these cells were cryopreserved and reported to have relatively low myoblast purity by the vendor. Freshly isolated myoblasts from patient biopsies^94^ or myoblasts subjected to rigorous purification^95^ may generate myotubes with higher levels of structural and functional maturity. Altering the culture protocol by utilizing multistep differentiation protocols with trophic factors^96^ or three-dimensional matrix support^63,97^ may also improve the maturation of myotubes generated from primary human myoblasts, but this was not explored in this study. Regardless of the source or culture procedure, primary human myoblasts are relatively inaccessible to many researchers, are generally collected in low quantities, have limited passage lifetimes, and are susceptible to patient-dependent variability,^98^ limiting scalability. Immortalized human myoblasts have also been generated,^99,100^ but these cells have not been commercialized and thus also remain relatively inaccessible.^15^ More recently, protocols for differentiating myoblasts from hiPSCs have been established,^49^ although they tend to suffer from low purity or yield^101^ and limited maturity,^102^ which will likely stunt NMJ formation. In contrast, primary chick myoblasts are easy to access and generate relatively mature muscle tissues, as evaluated in this study, and thus should be considered a suitable alternative for neuromuscular disease models. The non-human origin of chick muscle is somewhat mitigated by the integration of hiPSC-derived motor neurons, as many neuromuscular diseases are driven largely by dysfunction of the motor neurons and their inability to form and maintain NMJs.^1,3^ Thus, many patient- or mutation-specific features of neuromuscular diseases can likely be adequately captured by hiPSC-derived motor neurons co-cultured with chick muscle tissues. Collectively, these tradeoffs in myoblast source are critical to consider for engineering neuromuscular disease models and identifying which features of the muscle tissue, i.e., NMJ maturity or human relevance, are most relevant to the pathology of the disease of interest.

Three-dimensional muscle bundles derived from primary,^68^ immortalized,^103^ and hiPSC-derived^20^ myoblasts have also been co-cultured with motor neuron spheroids, leading to relatively advanced neuromuscular tissues. However, these constructs require large quantities of cells and technical expertise to fabricate and interrogate. Imaging also requires tissue sectioning or clearing,^104^ which requires considerable skill or time, respectively. Thus, the two-dimensional neuromuscular tissues engineered in this study have advantages in scalability, reproducibility, and imaging capabilities compared to three-dimensional tissue models.

In conclusion, muscle tissues engineered from chick myoblasts on micromolded gelatin hydrogels are relatively mature in structure and function and form advanced NMJs with hiPSC-derived motor neurons compared to previous *in vitro* approaches. By integrating hiPSC-derived motor neurons sourced from a variety of patients, these techniques for engineering neuromuscular tissues with advanced structure and function and rigorously interrogating them on a cellular and molecular level can be extended to improve neuromuscular disease modeling and drug development *in vitro*.

## METHODS

### Gelatin hydrogel substrate fabrication

The gelatin hydrogel fabrication process is summarized in Fig. S2. 150 mm polystyrene Petri dishes were covered with tape (Patco, 3900 R) and laser-cut into 260 mm^2^ hexagons to fit in 12-well plates using a 30 W Epilog Mini 24 Laser Engraver (100% speed, 25% power, 2500 Hz). Within each hexagon, circles were laser-cut into the tape (18% speed, 6% power, and 2500 Hz). For MTF substrates, two additional rectangular areas (4.1 × 9.9 mm^2^) were laser-cut into the tape. Circles of tape were removed such that only the edges of all substrates and rectangular areas of MTF substrates remained masked.

PDMS stamps with lines with 10 *μ*m width, 10 *μ*m spacing, and 2 *μ*m depth were fabricated using photolithography and soft lithography.^23,26,27^ Equal volumes of 20% w/v 175 g Bloom Type A porcine gelatin (Sigma, G2625) in ultrapure water at 65 °C and 8% Activa TI transglutaminase (TG) (Ajinomoto, 1002) in ultrapure water at 37 °C were combined and homogenized (30 s) and degassed (20 s) in a centrifugal mixer (Thinky USA, AR-100).^23^

Polystyrene hexagons were treated with plasma (Harrick Plasma, PDC-001-HP) in ambient air for ten minutes. Tape rectangles were removed from MTF substrates. Hydrogel solution was pipetted onto the polystyrene and PDMS stamps were slowly applied. Taped edges ensured consistency in hydrogel height.^23,52^ Hydrogels were incubated overnight at room temperature and then rehydrated with ultrapure water. Stamps and remaining tape were removed. To fabricate MTFs, hydrogels were dried for 30 min at room temperature. Two rows of four cantilevers (3.4 × 1.4 mm^2^, separated by 0.8 mm) were laser-cut twice (15% speed, 4% power, 2500 Hz and then 13% speed, 3% power, 2500 Hz) in the hydrogel above the un-activated rectangular regions. Substrates were rinsed in phosphate buffered saline and kept at 4 °C for up to a week. Before cell seeding, substrates were sterilized with a UVO Cleaner Model 342 (Jelight Company) for 1 min.

To measure hydrogel thickness, Alexa Fluor 546 conjugated fluorescent beads were embedded into hydrogels. The total height of fluorescence was quantified with a Nikon Eclipse Ti microscope at five locations per sample. To measure hydrogel elastic modulus, cylindrical samples (6 mm diameter) were fabricated and incubated in low glucose Dulbecco's Modified Eagle Medium (Gibco, 11885084) in a 37 °C, 5% CO_2_ incubator. DMEM was replaced every other day. After two weeks, compression testing until 30% strain was performed on samples using an Instron 5942 single column tabletop tester and Bluehill 3 testing software. Elastic modulus was defined as the slope of the linear region of the compressive stress–strain curves.

### Muscle cell and tissue culture

C2C12 (ATCC) and primary human skeletal myoblasts (Lonza) were thawed and cultured in growth media (Table S3) in T175 flasks. Thigh muscle tissues from day 10 chick embryos (AA Lab Eggs) were isolated and minced using forceps and scalpel.^24^ Four three-minute collagenase (Worthington LS004177, Lot 43K144303B) (1 mg/ml in Hank's Balanced Salt solution) digestions were performed at 37 °C, with mechanical dissociations by pipetting between digestions. Two 30-min pre-plating steps at 37 °C in T75 and T175 flasks were used to purify myoblasts. All myoblasts were expanded in T175 flasks in growth media and passaged using trypsin-EDTA solution at 80% confluence. Myoblasts were then seeded onto hydrogels (500 000 cells/substrate, C2C12 and human: passages 3–6, and chick: passages 0–3), cultured in growth media until confluence (3–4 days), and switched to differentiation media (Table S3). All media was refreshed every other day.

### Structural characterization of muscle tissues

Tissues were fixed using ice cold methanol for ten minutes and incubated with antibodies for sarcomeric ɑ-actinin (Sigma, A7811, dilution 1:200), followed by goat anti-mouse antibody conjugated to Alexa Fluor 546, α-bungarotoxin conjugated to Alexa Fluor 647, and 4′,6-diamidino-2-phenylindole (DAPI) (all 1:200 dilutions). Coverslips were mounted onto glass slides with ProLong gold antifade mountant (ThermoFisher Scientific, P36930) and stored at −20 °C. Widefield microscopy was performed using a Nikon Eclipse Ti microscope with 20× air and 60× oil objectives and an Andor Zyla sCMOS camera. Confocal microscopy was performed using a Confocal Module Nikon C2 with the same objectives. z-stacks were acquired (step size: 0.5 *μ*m) and maximum intensity projections were used for data analysis.

To quantify the number of nuclei, 24 fields of view per tissue were stitched (total area = 11.1 mm^2^) and cropped to reduce noise from edge effects or other abnormalities. CellProfiler (Broad Institute) was used to determine the number of nuclei per area based on intensity and size thresholding of DAPI signal. To quantify myogenic index, CellProfiler was used to generate a mask based on ɑ-actinin signal and determine the proportion of nuclei located in masked areas. To quantify sarcomere index,^105,106^ 2D fast Fourier transforms were performed using ImageJ on five randomly selected 50 *μ*m-wide myotube sections per tissue. The data from the transforms were collapsed radially to generate 1D power spectrum profiles, which were normalized to an integrated area of one. MATLAB curve fitting was used to divide the profile into aperiodic (decaying exponential) and periodic (sum of Gaussian functions) components. The fitted aperiodic component was subtracted from the total and the area under the periodic component was taken as the sarcomere index. Automated z-disc detection code^100^ was used to quantify sarcomere length from regions of interest. Myotube width was measured by averaging the widths of every myotube in five square fields of view (0.1 mm^2^), chosen randomly from the stitched image.

### Muscular thin film assay

Tissues on MTF substrates were transferred to a 35 mm Petri dish on a Nikon SMZ745T stereomicroscope and rinsed in 37 °C Tyrode's solution (5.0 mM HEPES, 1.0 mM magnesium chloride, 5.4 mM potassium chloride, 135.0 mM sodium chloride, 0.33 mM sodium phosphate, 1.8 mM calcium chloride, 5.0 mM glucose). Films were peeled using tweezers and platinum field stimulation electrodes were used to apply 20 V at 2 Hz or 20 Hz to induce twitch or tetanus contractions, respectively. Videos were recorded at 100 frames per second with a Basler acA640–120 *μ*m USB 3.0 camera. Custom ImageJ (NIH) and MATLAB (Mathworks) software was used to determine the radius of curvature of each MTF cantilever and calculate tissue stress using a modified Stoney's equation.^57,105,107^ Basal, twitch, and tetanus stresses were defined as the minimum stress observed during pacing, the average peak stress over eight contraction cycles paced at 2 Hz, and the average stress generated during one second stimulation at 20 Hz, respectively. The tetanus-to-twitch ratio was determined by dividing the tetanus stress by the twitch stress for each cantilever.

### Myotube co-culture with hiPSC-derived motor neurons

Healthy hiPSCs were procured from Coriell (ND03719, ND05280, ND00184, ND03231) and differentiated into motor neurons following published protocols.^12,85^ Briefly, hiPSCs were treated with small molecule cocktails that included CHIR99021, DMH1, and SB431542 to differentiate into OLIG2-positive motor neuron progenitors. These progenitors were removed from the culture surface with Accutase and seeded into non-tissue culture treated polystyrene dishes to form spheroidal aggregates. Cultures were further enriched through Notch inhibition (Compound E) and Hedgehog activation (purmorphamine). Motor neuron spheroids were then broken apart into small aggregates by slowly dissociating with a P1000 pipette until no chunks remained visible by eye. A homogenous sample of these aggregates was taken and further dissociated to obtain a reliable cell count. Finally, motor neurons were seeded dropwise at a density of roughly 750 000 neurons per substrate on top of myotubes undergoing differentiation for two to three days. All co-cultures were maintained in chick differentiation media supplemented with 10 *μ*m Rho-associated protein kinase inhibitor (Selleck, S1049), which was removed after one day, and 10 ng/ml *BDNF*, CNTF, and GDNF, which were removed after one week.^24,85^ Media was refreshed every other day.

### NMJ structural characterization

Tissues were fixed using ice cold methanol for ten minutes and incubated with antibodies for synapsin-1 (Cell Signaling Technology, D12G5, 1:200), followed by goat anti-chicken antibody conjugated to Alexa Fluor 488, goat anti-mouse antibody conjugated to Alexa Fluor 546 or α-bungarotoxin conjugated to Alexa Fluor 555, goat anti-mouse antibody conjugated to Alexa Fluor 633, and DAPI (all 1:200). Samples were imaged using the same confocal microscope and settings as described for monocultures.

The area and co-localization of synapsin-1 and bungarotoxin were analyzed with custom NIS Elements AR Analysis 5.02.00 macros.^34^ Motor neuron axons and clusters of acetylcholine receptors were defined by intensity thresholding of the synapsin-1 and bungarotoxin stains, respectively. For the synapsin-1 stain, area masks were created to fill holes. Data are reported as absolute area or proportion of the area in a field of view. Each data point represents two fields of view averaged per coverslip.

### NMJ electrophysiology

Muscle tissue cultures were rinsed and resuspended in Tyrode's solution with 20 *μ*m blebbistatin to prevent spontaneous or neuron-induced muscle contraction that would disrupt the measuring electrode with motion artifacts. Sharp electrode (electrode resistance between 10 and 20 MΩ) intracellular current-clamp recordings were performed in individual myotubes at room temperature with an Olympus BX61 WI microscope using a 40×/0.80 NA water-dipping objective and acquired using an Axoclamp 900 A amplifier, Digidata 1440 A acquisition system, and pClamp 10.5 software (Molecular Devices).^108^ Sweeps were digitized at 10 kHz and filtered at 1 kHz. Miniature excitatory postsynaptic potentials (mEPSPs) were recorded in the absence of stimulation for 5–10 min. Individual mEPSPs were selected manually by detecting signals over a set noise threshold that fit an mEPSP waveform. If present, muscle action potentials generated from spontaneous currents were ignored based on their characteristic waveform, which is quite distinct from mEPSPs.^11,109^ From at least six mEPSPs per myotube, amplitude, frequency, and rise and decay time constants (τ) were quantified. The resting membrane potential of each myotube was also quantified. Data were analyzed using Clampfit (Molecular devices), MiniAnalysis (Synaptosoft), Excel (Microsoft), and SigmaPlot (Systat) software. Each data point represents a recording from one myotube.

### RNAseq and bulk transcriptomic analysis

Engineered tissues were lysed using 1 ml of TRIzol reagent (Thermo Fisher Scientific) and RNA isolation was performed per the reagent manufacturer's protocol. Briefly, at 4 °C, lysate underwent two chloroform phase separation steps to isolate RNA from other cellular components. RNA was then precipitated with isopropyl alcohol and two 75% ethanol washes were performed to remove trace amounts of lysing agent and phenols to improve RNA purity. Total RNA was resuspended in 10 mM Trizma HCl (pH = 8.0) and stored at −80 °C prior to sequencing with an Illumina NovaSeq 6000 system (performed by Novogene Corporation, Inc.). Sequences (GEO Series accession No. GSE172606)^110^ were filtered and normalized with fragments per kilobase of transcript per million mapped reads—upper quartile (FPKM-UQ) normalization using Partek Flow Genomic Analysis software. Data were visualized through principal component and gene specific analysis with p = 0.05 and a fold change of 1.5 to define differentially expressed genes. Fold-change data from Partek Flow were uploaded to Qiagen Ingenuity Pathway Analysis (IPA) software with the same fold-change cutoffs to perform pathway analysis and generate predictive mechanistic network schematics. Specific networks were generated by overlaying imported fold-change data onto interaction networks from the library.

### Statistical analysis

All statistics and plots (except for RNAseq data) were generated using GraphPad Prism 7.04. Muscle monoculture data were analyzed using two-way analysis of variance (ANOVA) and Tukey's multiple comparisons test. For co-cultures, normal data, as evaluated with Shapiro–Wilk tests, were analyzed with either one-way ANOVA and compared with Tukey's multiple comparisons test or student's unpaired t-test, as appropriate. Data not normally distributed were compared with Mann–Whitney tests. Comparisons with p-values less than 0.05 were considered statistically significant. In figures, significance bars indicate statistical differences due to cell source or time point, but not both variables, to simplify data interpretation.

## SUPPLEMENTARY MATERIAL

See the supplementary material for full PCA analysis, gene expression fold-change data, complete formulations of cell culture media, acetylcholine receptor immunostaining of muscle tissues without hiPSC-derived motor neurons, and video of MTF twitch and tetanus contractions for each cell source after one and three weeks of differentiation.

## Data Availability

The data that support the findings of this study are available from the corresponding author upon reasonable request. Sequencing data discussed in this publication are openly available in NCBI's Gene Expression Omnibus, GEO Series Accession No. GSE172606 at https://www.ncbi.nlm.nih.gov/geo/query/acc.cgi?acc=GSE172606, Ref. 111.
